# Supplementary material for: Time-series transcriptome analysis identified differentially expressed genes in broiler chicken infected with mixed Eimeria species
Source: Front Genet. 2022 Aug 8;13:886781. doi: 10.3389/fgene.2022.886781 (PMC9393255; doi:10.3389/fgene.2022.886781)
Supplement: Supplementary file 2 [file DataSheet1.ZIP › 4dpi_KEGG.Gsea.1625100298584/KEGG_LYSOSOME.html]

Details for gene set KEGG\_LYSOSOME[GSEA]

|  || Dataset | TMM\_4dpi\_gct\_format\_4dpi\_gct\_format.Class\_4dpi.cls #PC\_versus\_NC.Class\_4dpi.cls #PC\_versus\_NC\_repos |
| Phenotype | Class\_4dpi.cls#PC\_versus\_NC\_repos |
| Upregulated in class | 0 |
| GeneSet | KEGG\_LYSOSOME |
| Enrichment Score (ES) | -0.4145537 |
| Normalized Enrichment Score (NES) | -1.7399852 |
| Nominal p-value | 0.0 |
| FDR q-value | 0.08696112 |
| FWER p-Value | 0.3016 |
Table: GSEA Results Summary

  

Fig 1: Enrichment plot: KEGG\_LYSOSOME      
 Profile of the Running ES Score & Positions of GeneSet Members on the Rank Ordered List

  

| SYMBOL | TITLE | RANK IN GENE LIST | RANK METRIC SCORE | RUNNING ES | CORE ENRICHMENT || 1 | LIPA | na | 288 | 1.188 | 0.0077 | No |
| 2 | ABCB9 | na | 430 | 1.035 | 0.0236 | No |
| 3 | AP1S3 | na | 763 | 0.778 | 0.0165 | No |
| 4 | NPC1 | na | 1035 | 0.659 | 0.0115 | No |
| 5 | CLTC | na | 1107 | 0.636 | 0.0225 | No |
| 6 | AP4E1 | na | 1135 | 0.628 | 0.0371 | No |
| 7 | DNASE2B | na | 2001 | 0.423 | -0.0242 | No |
| 8 | AP4S1 | na | 2277 | 0.378 | -0.0371 | No |
| 9 | LAMP1 | na | 2290 | 0.377 | -0.0280 | No |
| 10 | AP3B1 | na | 2582 | 0.337 | -0.0434 | No |
| 11 | CLTA | na | 2904 | 0.296 | -0.0625 | No |
| 12 | AP1S2 | na | 3096 | 0.269 | -0.0713 | No |
| 13 | LAPTM4A | na | 3428 | 0.228 | -0.0930 | No |
| 14 | AP3S1 | na | 3449 | 0.227 | -0.0886 | No |
| 15 | GGA3 | na | 3478 | 0.223 | -0.0849 | No |
| 16 | CLTCL1 | na | 3572 | 0.210 | -0.0871 | No |
| 17 | AP1G1 | na | 3737 | 0.195 | -0.0957 | No |
| 18 | SGSH | na | 3838 | 0.183 | -0.0991 | No |
| 19 | CTSS | na | 3868 | 0.181 | -0.0967 | No |
| 20 | CTSK | na | 4067 | 0.162 | -0.1090 | No |
| 21 | ATP6V0A4 | na | 4081 | 0.161 | -0.1058 | No |
| 22 | AP1M1 | na | 4558 | 0.119 | -0.1426 | No |
| 23 | ATP6V0D2 | na | 4847 | 0.094 | -0.1643 | No |
| 24 | SLC11A2 | na | 4980 | 0.080 | -0.1732 | No |
| 25 | TCIRG1 | na | 5120 | 0.069 | -0.1830 | No |
| 26 | CLTB | na | 5444 | 0.041 | -0.2091 | No |
| 27 | SORT1 | na | 5494 | 0.037 | -0.2122 | No |
| 28 | CTSD | na | 5549 | 0.031 | -0.2159 | No |
| 29 | LAPTM4B | na | 5568 | 0.029 | -0.2166 | No |
| 30 | ATP6V1H | na | 5862 | 0.005 | -0.2411 | No |
| 31 | NAGPA | na | 5990 | -0.005 | -0.2516 | No |
| 32 | CTNS | na | 6039 | -0.009 | -0.2554 | No |
| 33 | GNPTG | na | 6118 | -0.016 | -0.2615 | No |
| 34 | AP3M2 | na | 6170 | -0.020 | -0.2653 | No |
| 35 | GUSB | na | 6218 | -0.024 | -0.2686 | No |
| 36 | CTSG | na | 6231 | -0.025 | -0.2689 | No |
| 37 | ATP6V0D1 | na | 6337 | -0.033 | -0.2768 | No |
| 38 | ATP6V0C | na | 6344 | -0.033 | -0.2764 | No |
| 39 | CD164 | na | 6460 | -0.044 | -0.2849 | No |
| 40 | GGA2 | na | 6730 | -0.065 | -0.3058 | No |
| 41 | CTSB | na | 6909 | -0.080 | -0.3186 | No |
| 42 | GNS | na | 6916 | -0.081 | -0.3169 | No |
| 43 | AP1B1 | na | 7055 | -0.094 | -0.3260 | No |
| 44 | ATP6V0A2 | na | 7233 | -0.112 | -0.3378 | No |
| 45 | HGSNAT | na | 7641 | -0.147 | -0.3681 | No |
| 46 | ASAH1 | na | 7648 | -0.148 | -0.3646 | No |
| 47 | ACP2 | na | 7947 | -0.174 | -0.3850 | No |
| 48 | GAA | na | 8167 | -0.194 | -0.3982 | No |
| 49 | HEXB | na | 8235 | -0.202 | -0.3984 | No |
| 50 | ATP6V0A1 | na | 8243 | -0.203 | -0.3935 | No |
| 51 | NPC2 | na | 8394 | -0.218 | -0.4003 | No |
| 52 | ABCA2 | na | 8565 | -0.237 | -0.4082 | Yes |
| 53 | CD63 | na | 8573 | -0.238 | -0.4024 | Yes |
| 54 | HEXA | na | 8634 | -0.243 | -0.4009 | Yes |
| 55 | GGA1 | na | 8638 | -0.244 | -0.3947 | Yes |
| 56 | AP3M1 | na | 8693 | -0.250 | -0.3925 | Yes |
| 57 | IDS | na | 8739 | -0.254 | -0.3895 | Yes |
| 58 | IDUA | na | 8780 | -0.259 | -0.3859 | Yes |
| 59 | GLB1 | na | 8873 | -0.270 | -0.3863 | Yes |
| 60 | CTSO | na | 8999 | -0.283 | -0.3893 | Yes |
| 61 | AP3D1 | na | 9113 | -0.297 | -0.3908 | Yes |
| 62 | PPT1 | na | 9166 | -0.304 | -0.3870 | Yes |
| 63 | GALC | na | 9197 | -0.308 | -0.3813 | Yes |
| 64 | AP3B2 | na | 9338 | -0.327 | -0.3842 | Yes |
| 65 | MANBA | na | 9358 | -0.330 | -0.3770 | Yes |
| 66 | MFSD8 | na | 9380 | -0.333 | -0.3698 | Yes |
| 67 | ENTPD4 | na | 9393 | -0.335 | -0.3619 | Yes |
| 68 | SUMF1 | na | 9403 | -0.336 | -0.3536 | Yes |
| 69 | LGMN | na | 9449 | -0.342 | -0.3482 | Yes |
| 70 | ARSA | na | 9476 | -0.344 | -0.3412 | Yes |
| 71 | MCOLN1 | na | 9485 | -0.346 | -0.3326 | Yes |
| 72 | M6PR | na | 9616 | -0.364 | -0.3337 | Yes |
| 73 | CTSH | na | 9822 | -0.389 | -0.3405 | Yes |
| 74 | GNPTAB | na | 9863 | -0.394 | -0.3333 | Yes |
| 75 | AP4B1 | na | 10070 | -0.424 | -0.3392 | Yes |
| 76 | FUCA1 | na | 10147 | -0.437 | -0.3339 | Yes |
| 77 | SLC11A1 | na | 10160 | -0.438 | -0.3231 | Yes |
| 78 | CTSA | na | 10299 | -0.460 | -0.3224 | Yes |
| 79 | HYAL1 | na | 10313 | -0.462 | -0.3111 | Yes |
| 80 | ARSB | na | 10339 | -0.468 | -0.3006 | Yes |
| 81 | IGF2R | na | 10545 | -0.507 | -0.3043 | Yes |
| 82 | CTSZ | na | 10667 | -0.532 | -0.3001 | Yes |
| 83 | AGA | na | 10740 | -0.547 | -0.2915 | Yes |
| 84 | NAGA | na | 10744 | -0.548 | -0.2771 | Yes |
| 85 | ATP6V0B | na | 10748 | -0.548 | -0.2626 | Yes |
| 86 | CTSV | na | 10774 | -0.554 | -0.2499 | Yes |
| 87 | LAMP3 | na | 10868 | -0.575 | -0.2423 | Yes |
| 88 | SCARB2 | na | 10909 | -0.584 | -0.2300 | Yes |
| 89 | LAPTM5 | na | 11094 | -0.634 | -0.2284 | Yes |
| 90 | PSAP | na | 11105 | -0.639 | -0.2121 | Yes |
| 91 | PLA2G15 | na | 11204 | -0.664 | -0.2025 | Yes |
| 92 | ACP5 | na | 11217 | -0.670 | -0.1856 | Yes |
| 93 | AP3S2 | na | 11266 | -0.688 | -0.1712 | Yes |
| 94 | SMPD1 | na | 11304 | -0.706 | -0.1554 | Yes |
| 95 | NAGLU | na | 11327 | -0.712 | -0.1381 | Yes |
| 96 | CTSC | na | 11374 | -0.736 | -0.1222 | Yes |
| 97 | SLC17A5 | na | 11380 | -0.740 | -0.1028 | Yes |
| 98 | ATP6AP1 | na | 11499 | -0.807 | -0.0911 | Yes |
| 99 | TPP1 | na | 11520 | -0.819 | -0.0708 | Yes |
| 100 | GLA | na | 11566 | -0.849 | -0.0518 | Yes |
| 101 | CLN5 | na | 11599 | -0.875 | -0.0311 | Yes |
| 102 | LAMP2 | na | 11682 | -0.945 | -0.0126 | Yes |
| 103 | ARSG | na | 11938 | -1.493 | 0.0060 | Yes |
Table: GSEA details [plain text format]

  

Fig 2: KEGG\_LYSOSOME      
 Blue-Pink O' Gram in the Space of the Analyzed GeneSet

  

Fig 3: KEGG\_LYSOSOME: Random ES distribution      
 Gene set null distribution of ES for **KEGG\_LYSOSOME**
